# Using the Comprehensive Complication Index to Assess the Impact of Neoadjuvant Chemoradiotherapy on Complication Severity After Esophagectomy for Cancer

**DOI:** 10.1245/s10434-016-5291-3

**Published:** 2016-06-14

**Authors:** Nina Nederlof, Annelijn E. Slaman, Pieter van Hagen, Ate van der Gaast, Ksenija Slankamenac, Suzanne S. Gisbertz, Jan J. B. van Lanschot, Bas P. L. Wijnhoven, Mark I. van Berge Henegouwen, CROSS-Study Group, J. J. Bonenkamp, O. R. C. Busch, M. A. Cuesta, G. A. P. Nieuwenhuijzen, J. T. M. Plukker, E. J. Spillenaar Bilgen, H. W. Tilanus

**Affiliations:** 1 Department of Surgery, Erasmus University Medical Center, Rotterdam, The Netherlands; 2 Department of Radiation Oncology, Erasmus University Medical Center, Rotterdam, The Netherlands; 3 Department of Surgery G4-115, Academic Medical Center, Amsterdam, The Netherlands; 4 University Hospital Zurich, Zurich, Switzerland

## Abstract

**Background:**

Neoadjuvant chemoradiotherapy (nCRT) followed by surgery for patients with esophageal or junctional cancer has become a standard of care. The comprehensive complication index (CCI) has recently been developed and accounts for all postoperative complications. Hence, CCI better reflects the burden of all combined postoperative complications in surgical patients than the Clavien–Dindo score alone, which incorporates only the most severe complication. This study was designed to evaluate the severity of complications in patients treated with nCRT followed by esophagectomy versus in patients who underwent esophagectomy alone using the comprehensive complication index.

**Study-design:**

All patients included in the CROSS trial—a randomized, clinical trial on the value of nCRT followed by esophagectomy—were included. Complications were assessed and graded using the Clavien–Dindo classification. CCI was derived from these scores, using the CCI calculator available online (www.assessurgery.com). CCI of patients who underwent nCRT followed by surgery was compared with the CCI of patients who underwent surgery alone.

**Results:**

In both groups 161 patients were included. The median (and interquartile range) CCI of patients with nCRT and surgery was 26.22 (17.28–42.43) versus 25.74 (8.66–43.01) in patients who underwent surgery alone (*p* = 0.58). There also was no difference in CCI between subgroups of patients with anastomotic leakage, pulmonary complications, cardiac complications, thromboembolic events, chyle leakage, and wound infections.

**Conclusions:**

Neoadjuvant chemoradiotherapy according to CROSS did not have a negative impact on postoperative complication severity expressed by CCI compared with patients who underwent surgery alone for potentially curable esophageal or junctional cancer.

Esophageal cancer remains one of the most common cancers worldwide.^1^ Treatment for patients with potentially curable esophageal cancer is an esophagectomy with gastric tube reconstruction. Meta-analyses of randomized, controlled trials comparing neoadjuvant chemoradiotherapy plus surgery to surgery alone showed that multimodality treatment improves overall survival, but side-effects (e.g., radiofibrosis, suppressed immune function, impaired nutritional and hematological status) could increase morbidity and mortality after esophagectomy.^2^–^17^

The largest, published, randomized clinical trial on the value of neoadjuvant chemoradiotherapy (CROSS-trial) also showed a survival benefit.^8^ Importantly, there was no difference in the frequency of complications and postoperative mortality between the patients who were treated with neoadjuvant chemoradiotherapy followed by surgery and the patients who underwent surgery alone.

In the past decades, not only the frequency but also the severity of postoperative complications has become an important quality measure in surgical studies. Also, patients’ reported grading of complications gives a better insight into the burden of a complicated postoperative course. Therefore, several severity-scoring systems have been developed.^18^–^22^ A novel and validated scoring system is the Comprehensive Complication Index (CCI).^20^,^23^ CCI summarizes the frequency, severity, and patient’s rating of complications by using the adopted “operating risk index” in a single score that ranges between 0 (no complication) and 100 (death) based on the established Clavien–Dindo classification.^22^ Therefore, it accounts for the whole burden of all complications. A recent study showed that CCI is a sensitive method that is superior to traditional endpoints, because it summarizes the whole burden of postoperative complications to the patient with respect to complications.^20^ Whereas traditional endpoints showed no significant differences for incidence of postoperative complications within the CROSS trial, the current study was designed to evaluate the overall effect of neoadjuvant chemoradiotherapy on the severity of postoperative complications and the overall burden in patients of the CROSS trial. Therefore, the CCI was compared between patients with esophageal or esophagogastric junction cancer who underwent chemoradiotherapy plus surgery versus patients who underwent surgery alone.

## Patients and Methods

Patients with esophageal cancer or cancer of the esophagogastric junction (cT1-4aN0-3M0) who underwent a curative surgical resection of the esophagus and who participated in the CROSS trial were selected from the study database. The CROSS trial is a multicenter, randomized, controlled trial that compared overall survival for patients who were treated with neoadjuvant chemoradiotherapy followed by esophagectomy and the patients who underwent esophagectomy alone. The inclusion and exclusion criteria as well as staging procedures have been described previously.^24^ As the study focuses on complication severity after esophagectomy, patients who did not undergo resection were removed from the study cohort.

### Complications

Complications were defined using the complete and commonly applicable National Cancer Institute’s Common Terminology Criteria for Adverse Events, 4.0.^25^ Because these criteria do not provide a definition of anastomotic leakage, the definition according to Bruce et al.^26^ was used: drainage of saliva or gastrointestinal content from the surgical join between the oesophagus and gastric tube. The luminal contents may emerge externally or internally or may be collected near the anastomosis with or without systemic complications. Only complications within 30 days after the operation and/or during hospital stay were assessed.

### CCI

The CCI is a complication index introduced by Slankamenac et al.^23^ in 2013 and is based on the Clavien–Dindo classification^22^ (Appendix). In the development of the CCI, data on common postoperative complications were gathered and rated by both patients and physicians. By this method, each complication is validated and given a fixed number and also includes patient’s perspective about the severity. After this, a score is calculated for each grade in the Clavien–Dindo classification. To calculate the CCI, all complications that a patient develops after surgery are summarized and computed through the operation risk index approach (commonly used in economics). This can be done easily and free of charges at www.assessurgery.com. The final index yields a score from 0 (no complication) to 100 (death).^27^

To investigate whether postoperative complication severity is influenced by neoadjuvant treatment, the severity of all combined complications was measured using the CCI. Based on results in earlier studies of patients who underwent esophageal cancer surgery in which specific complications have shown an increase in incidence, six subgroups were formed in this study. For example, some studies show influence of neoadjuvant treatment on pulmonary complications, due to the radiation field. In subgroup 2, patients with pulmonary complications are compared. Only patients with the specific complication were used to calculate the specific complication CCI.

### Grading of Complications

We used the original database of the CROSS study in which postoperative complications were scored by data managers in each participating center. Cross checking of these complications and grading every complication according to the Clavien–Dindo classification was done by one of the authors (NN). The CCI was calculated afterwards. In addition, for each patient the traditional endpoints, the total number of complications, the presence of any complication (yes/no) and the most severe complications (≥IIIb according to the Clavien–Dindo classification) were assessed.

### Treatment

As previously described,^24^ patients randomized to neoadjuvant chemoradiotherapy underwent five weekly cycles of chemoradiotherapy (carboplatin/paclitaxel with 41.1 Gy concurrent radiotherapy) followed by surgery, preferably within 4–6 weeks of completion. Patients randomized to the surgery alone arm underwent esophagectomy as soon as possible.

### Statistical Analysis

Adjustment for possible confounders was not necessary, because the data were controlled for confounding by randomization. Baseline continuous data were described as means with standard deviation or, in case of a not-normally distributed variable, with the median and interquartile range. Normal distribution was calculated using the Kolmogorov–Smirnov test. Groups were compared using the nonparametric Mann–Whitney *U* test. For cross-tabulations, Pearson’s chi-square test with continuity correction was used. All statistical analyses were performed on the statistical package SPSS 22.0 (SPSS Inc., Chicago, IL). *p* < 0.05 two-sided was considered statistically significant.

## Results

**Fig. 1:**
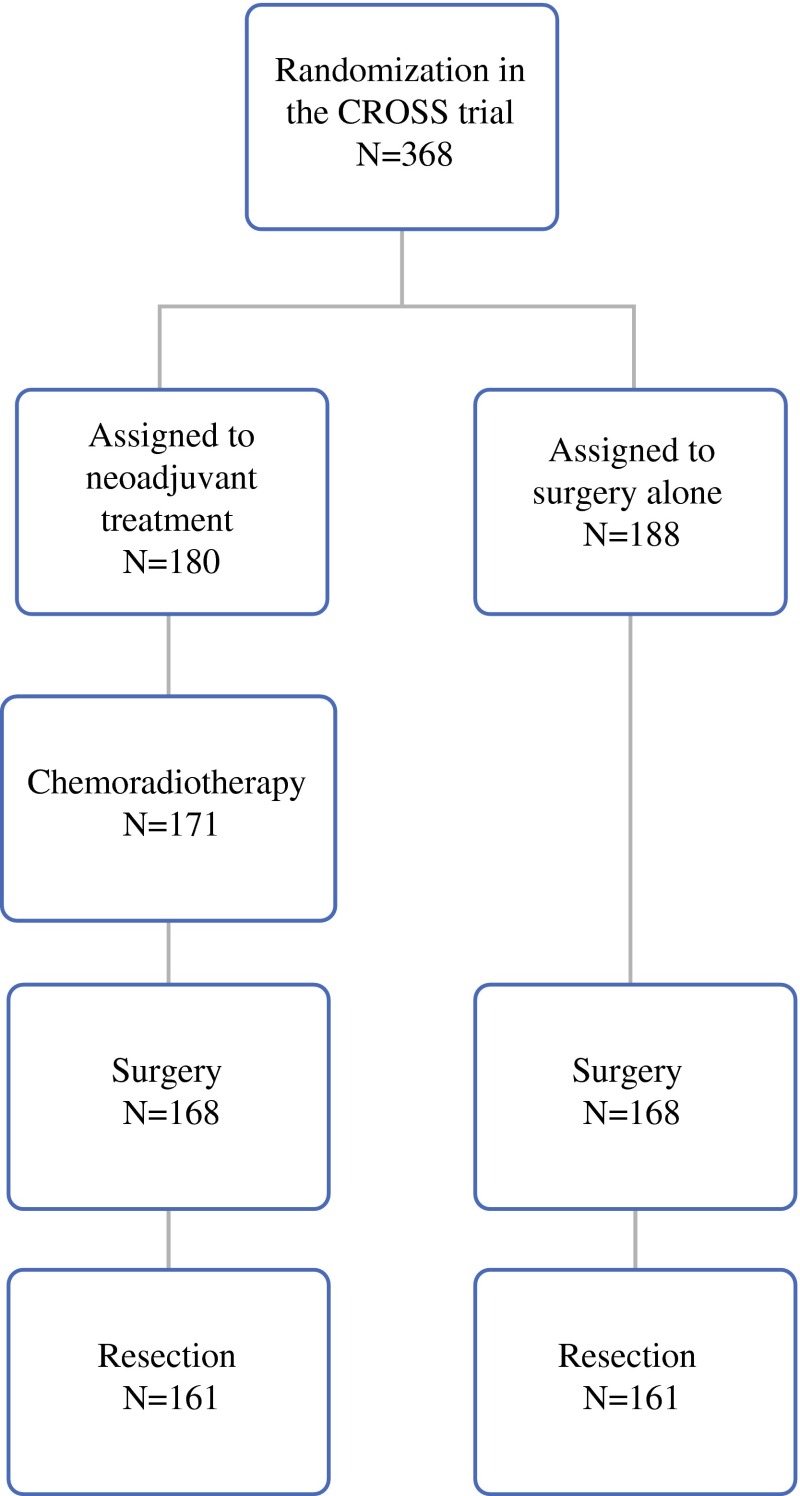
Flowchart patients

Of the 368 patients randomized in the CROSS trial, 322 patients were included in the present study.^8^ An overview of inclusion and exclusion of patients in the present study is shown in Fig. 1. Patient’s characteristics, including age, sex, comorbidity, and surgical approach, were similar between both groups (Table 1). More R0 resections were performed in patients who received nCRT before esophagectomy (*p* < 0.001). In patients who were analyzed in the current study (*n* = 322), the combined treatment group 136 (85 %) patients developed at least one complication versus 125 (78 %) the surgery alone group (*p* = 0.13; Table 2).

**Table 1:** Patient and tumor characteristics

	nCRT and surgery (161)	Surgery alone (161)	*p* value
Age (year) median [range]	60 [37–76]	60 [36–76]	0.72
Sex (M:F)	129:34	123:38	0.41
WHO performance status^a^			
0	27	20	0.28
1	134	140	0.34
Comorbidity			
Cardiovascular	45 (28 %)	40 (25 %)	0.48
Respiratory	17 (11 %)	19 (12 %)	0.69
Diabetes mellitus	14 (9 %)	11 (7 %)	0.55
Histology			
Squamous cell carcinoma	37	37	1.0
Adenocarcinoma	121	120	1.0
Undifferentiated carcinoma	3	4	1.0
Tumor site			
Proximal esophagus	2 (1 %)	3 (2 %)	1.0
Mid esophagus	24 (15 %)	16 (10 %)	0.23
Distal esophagus	112 (70 %)	123 (76 %)	0.20
Gastroesophageal junction	23 (14 %)	17 (12 %)	0.40
Mortality			
30-day	3 (2 %)	4 (3 %)	1.00
In-hospital	5 (3 %)	6 (4 %)	0.99
Surgical approach			
Transhiatal esophagectomy	72 (45 %)	72 (45 %)	1.0
Transthoracic esophagectomy	89 (55 %)	87 (54 %)	0.91
Resection with tumour-free margins p(R0)	148 (92 %)	111 (69 %)	<0.001

Percentages may not add up to 100 because of rounding

*WHO* World Health Organization; *nCRT* neoadjuvant chemoradiotherapy

^a^WHO performance status scores are on a scale of 0–5, with lower numbers indicating better performance status; 0 indicates fully active, and 1 unable to carry out heavy physical work

**Table 2:** Frequencies of Clavien–Dindo grades and postoperative complications in patients of the current study

	nCRT and surgery (*n* = 161)	Surgery alone (*n* = 161)	*p* value
Any complication	136 (85 %)	125 (78 %)	0.13
Grade I complication	70 (43 %)	79 (49 %)	0.37
Grade II complication	90 (56 %)	85 (53 %)	0.65
Grade IIIa complication	58 (36 %)	52 (32 %)	0.56
Grade IIIb complication	25 (13 %)	28 (15 %)	0.76
Grade IVa complication	28 (15 %)	33 (20 %)	0.57
Grade IVb complication	3 (2 %)	6 (3 %)	0.50
Grade V complication	5 (3 %)	6 (3 %)	1.00
Subgroup 1: Anastomotic leakage^a^	37 (23 %)	49 (30 %)	0.16
Subgroup 2: Pulmonary complications^b^	81 (50 %)	82 (50 %)	1.00
Subgroup 3: Cardiac complications^c^	34 (21 %)	23 (14 %)	0.57
Subgroup 4: Thromboembolic events	6 (3 %)	4 (2 %)	1.00
Subgroup 5: Chyle leakage^d^	16 (10 %)	11 (7 %)	0.41
Subgroup 6: Wound infections	18 (11 %)	21 (13 %)	0.60
Anastomotic leakage	37 (23 %)	49 (30 %)	0.16
Leakage requiring surgical intervention	8 (4 %)	6 (3 %)	0.59
Pneumonia	49 (30 %)	40 (21 %)	0.32
Atelectasis	17 (11 %)	22 (14 %)	0.49
Empyema	14 (9 %)	25 (16 %)	0.09
Pneumothorax	10 (6 %)	14 (9 %)	0.52
Respiratory insufficiency	29 (15 %)	33 (20 %)	0.67
Reintubation	33 (20 %)	33 (20 %)	1.00
Thromboembolism	6 (3 %)	4 (2 %)	0.75
Cardiac arrhythmia	30 (20 %)	22 (12 %)	0.29
Myocardial infaction	0 (0 %)	1 (1 %)	1.00
Cardiac decompensation	4 (2 %)	0 (0 %)	0.13
Mediastinitis	6 (3 %)	11 (7 %)	0.32
Chylothorax	16 (10 %)	11 (7 %)	0.41
Vocal cord palsy	19 (12 %)	12 (7 %)	0.66
Wound infection neck	9 (6 %)	6 (3 %)	0.60
Wound infection thorax	0 (0 %)	9 (6 %)	0.007
Wound infection abdomen	9 (6 %)	6 (3 %)	0.60
Renal failure	4 (2 %)	1 (1 %)	0.37
Sepsis	7 (4 %)	10 (6 %)	0.62
Multi-organ failure	0 (0 %)	4 (2 %)	0.13
Readmittance ICU	30 (19 %)	27 (17 %)	0.66

Adverse events were graded according to the National Cancer Institute’s Common Terminology Criteria for Adverse Events, version 4.0

*nCRTS* neoadjuvant chemoradiotherapy

^a^Anastomotic leakage was defined as: drainage of saliva or gastrointestinal content from the surgical join between the oesophagus and gastric tube. The luminal contents may emerge externally or internally, or may be collected near the anastomosis with or without systemic complications

^b^Pulmonary complications were pneumonia (isolation of pathogen from sputum culture and a new or progressive infiltrate on chest radiograph), serious atelectasis (lobar collapse on chest radiograph), pneumothorax (collection of air between the visceral and parietal pleural surfaces, requiring drainage), pleural effusion (collection of fluid between the visceral and parietal pleural surfaces, requiring drainage), pulmonary embolus (embolus detected on spiral CT or a ventilation–perfusion mismatch on a lung scintigram), and acute respiratory failure (partial pressure of arterial oxygen <60 mm Hg while breathing ambient air)

^c^Cardiac complications were arrhythmia (any change in rhythm on the electrocardiogram, requiring treatment), myocardial infarction (two or three of the following: previous myocardial infarction, electrocardiographic changes suggesting myocardial infarction, or enzyme changes suggesting myocardial infarction), cardiac decompensation and left ventricular failure (marked pulmonary edema on a chest radiograph)

^d^Chylothorax was recorded when elevated levels of triglycerides in intrathoracic fluid [>1 mmol l^−1^ (89 mg per deciliter)] were found. Mediastinitis was scored when reported by the local investigator

Grade I complications were seen in 43 % of patients after neoadjuvant chemoradiotherapy plus surgery versus 49 % of patients after surgery alone (*p* = 0.37). There also was no statistically significant difference for grade II-grade V complications (Table 2).

Analyses in six subgroups showed that respiratory complications, i.e., pneumonia were the most common (30 vs. 21 %, *p* = 0.32), followed by anastomotic leakage (23 vs. 30 %, *p* = 0.13) and cardiac arrhythmias (20 vs. 12 %, *p* = 0.29). Significantly more infections of the chest wound were found in patients with neoadjuvant treatment who underwent a transthoracic esophagectomy (0 vs. 6 %, *p* = 0.007). The incidence of all other complications was not significantly different between the two groups.

There was no statistically significant difference in the CCI between both groups. Median CCI in the combined treatment group was 26.22 (IQR 17.28–42.43) compared with 25.74 (IQR 8.66–43.01) in the surgery alone group (*p* = 0.58; Table 3).

**Table 3:** Comprehensive complication Index computed for the whole study group as well as subgroups of common postoperative complications

	CRTx and surgery	Surgery alone	*p* value
CCI (whole group; *N* = 322)	26.22 (17.28–42.43)	25.73 (8.66–43.01)	0.58
CCI patients with anastomotic leakage (*N* = 86)^a^	8.66 (8.66–33.73)	8.66 (8.66–33.73)	0.78
CCI patients with pulmonary complications (*N* = 163)^b^	20.92 (20.92–42.43)	20.92 (20.92–42.43)	0.59
CCI patients with cardiac complications (*N* = 57)^c^	20.92 (20.92–20.92)	20.92 (20.92–20.92)	0.64
CCI patients with thromboembolic events (*N* = 10)^d^	20.92 (20.92–20.92)	20.92 (20.92–20.92)	1.0
CCI patients with chyle leak (*N* = 27)^e^	8.66 (8.66–20.92)	14.79 (8.66–31.85)	0.65
CCI patients with wound infections (*N* = 39)^f^	8.66 (8.66–8.66)	8.66 (8.66–8.66)	0.93

CCI for the whole group was computed on all patients. CCI of subgroups were calculated only in patients with the specific complication, to compare the severeness of the specific complications between groups

Values are shown as median with interquartile range and *p* value

^a^Anastomotic leakage was defined as: drainage of saliva or gastrointestinal content from the surgical join between the oesophagus and gastric tube. The luminal contents may emerge externally or internally, or may be collected near the anastomosis with or without systemic complications

^b^Pulmonary complications were pneumonia (isolation of pathogen from sputum culture and a new or progressive infiltrate on chest radiograph), serious atelectasis (lobar collapse on chest radiograph), pleural effusion (collection of fluid between the visceral and parietal pleural surfaces, requiring drainage) and acute respiratory failure (partial pressure of arterial oxygen <60 mm Hg while breathing ambient air)

^c^Cardiac complications were arrhythmia (any change in rhythm on the electrocardiogram, requiring treatment), myocardial infarction (two or three of the following: previous myocardial infarction, electrocardiographic changes suggesting myocardial infarction, or enzyme changes suggesting myocardial infarction), cardiac decompensation and left ventricular failure (marked pulmonary edema on a chest radiograph)

^d^Thromboembolic events were defined as a deep venous thrombosis (shown on echo) or pulmonary embolus (embolus detected on spiral CT or a ventilation–perfusion mismatch on a lung scintigram)

^e^Chylothorax was recorded when elevated levels of triglycerides in intrathoracic fluid [>1 mmol l^−1^ [89 mg dl^−1^)] were found

^f^Wound infections were defined as redness, inflammation, with extravasation of pus after drainage

In subgroup analyses of the specific complications, CCI for patients who underwent neoadjuvant chemoradiotherapy and developed an anastomotic leak was not statistically different from patients who underwent surgery alone: 8.66 [8.66–33.73] vs. 8.66 [8.66–33.73] (*p* = 0.78). The same was true for the other subgroups with patients who developed pulmonary or cardiac complications, thromboembolic event, chyle leakage, or wound infection (Table 3).

## Discussion

The Dutch CROSS study showed an absolute 5-years survival benefit of 13 % for patients who underwent neoadjuvant chemoradiotherapy followed by an esophagectomy for esophageal or esophagogastric cancer. Hence, neoadjuvant chemoradiotherapy is nowadays widely used in clinical practice. However, it is important to consider the possible harm of neoadjuvant chemoradiotherapy, because trials frequently focus on the benefit of a treatment.^28^–^30^ This may be caused by a lack of sensitive outcome parameters, by underreporting, and by the strict inclusion criteria of trials that are frequently broadened after closure of the trial and the specifics of positive results. Also, sample sizes often are rather small, masking the incidence of selectively rare but potentially serious complications. This study used the novel outcome measure for postoperative complicated course (CCI) to compare the additive impact of neoadjuvant chemoradiotherapy on the severity of complications in patients after esophagectomy, as the incidence of complications is already reported in the CROSS study. Our results show neither a significant difference in CCI between both groups nor in the incidence of specific common complications.

The benefit of neoadjuvant treatment has been a topic of many studies but the harm has been described less extensively. The Cochrane review, published in 2010, demonstrates that postoperative complications often are ill described or missing at all.^7^,^31^ Therefore, in their meta-analysis no overall complication rate could be calculated. In a retrospective study published by Morita et al. containing 686 patients, the total number of complications, as well as pulmonary complications and anastomotic leakage developed more frequently in patients with neoadjuvant treatment in comparison with patients without neoadjuvant treatment.^32^ Bosch et al. confirmed an increase in cardiopulmonary complications in the neoadjuvant treatment group (pneumonia and cardiac arrhythmias).^16^ Merrit et al., in a retrospective cohort study of 138 patients, showed no increase in postoperative morbidity and mortality but concluded that major postoperative complications are rather due to surgical technique and preoperative morbidity rather than neoadjuvant therapy.^10^ Furthermore, Kelley et al. performed a prospective trial in 2004, which showed no significantly higher complication rate in patients with preoperative chemoradiotherapy.^13^ In a study of 40 patients by Bagheri et al., respiratory complications were closely analyzed, and although there was a significant correlation between the number of microorganisms in the sputum and difficulty in weaning, there was no correlation found between neoadjuvant treatment and pulmonary complications.^15^ Several meta-analyses showed a decrease in mortality without any proof of a decrease in postoperative complications, but most trials failed to produce information about postoperative complications.^4^,^6^,^9^,^17^ Greer et al. found no difference in their meta-analysis and concluded that there was a need for large, randomized trials.^5^

With the recently developed sensitive comprehensive complication index, it is possible to take the severity of all complications into consideration, thus improving the accuracy of reporting the impact of all side effects combined.^20^,^23^ The CCI has been validated already in different surgical trials, showing its value. The CCI incorporates patients’ opinion on a complication, as well as the physicians’ opinion. It also takes into account low-grade complications, which are normally not considered an endpoint but adds up to the patients’ postoperative experience. Additionally, the CCI can be used to compare the severity of a specific complication (i.e., anastomotic leakage) between different patient groups (Table 3).

There are several limitations to the current study. Because our study included patients from seven participating hospitals, it may be possible that there is some difference in reporting and treatment of complications. All complications were reviewed by one of the authors to preserve uniformity in application of the Clavien–Dindo classification. In the Netherlands, the transference to the Medium or Intensive Care Unit for more intensive monitoring of the patients is relatively low, which in the Clavien–Dindo system directly results in a grade IV complication but is not always accompanied by organ failure. The difference in complications scored in the CROSS trial differ because of the difference between the Clavien–Dindo classification and the CCI. In the CROSS study, only the most severe complication counted. This study only reports early complications, within 30 days and/or within hospital admission. Later complications, e.g., stenosis or complications due to recurrence, were not included. Another possible limitation of this study is that postoperative complications were not the primary endpoint of the CROSS trial. The study was powered to show a difference in overall survival; therefore, the sample size of this study might be too small to show differences in rare complications. However, as described by Slankamenac et al., when using the CCI as opposed to the original Clavien–Dindo classification as an endpoint, meaningful comparison can be obtained with smaller sample sizes.^20^

The CCI can be used as a tool to monitor postoperative recovery in a detailed and structured way. Because all data in the present study were prospectively registered, this study shows a realistic view of postoperative complications in patients with cancer of the esophageal and esophagogastric junction. This study shows that the frequency of complications described in patients extracted from CROSS trial is similar in the two groups; and the outcome of specific complications in the two groups is similar. Neoadjuvant chemoradiotherapy does not show a negative impact on the overall postoperative morbidity as expressed by the CCI compared with patients who underwent surgery alone for potentially curable esophageal or esophagogastric junctional cancer.

## Appendix

See Table 4.

**Table 4:** Clavien–Dindo classification

Grade	Description
Grade I	Any deviation from the normal postoperative course without the need for pharmacological treatment or surgical, endoscopic, and radiological interventions Allowed therapeutic regimens are: drugs as antiemetics, antipyretics, analgetics, diuretics, electrolytes, and physiotherapy. This grade also includes wound infections opened at the bedside
Grade II	Requiring pharmacological treatment with drugs other than such allowed for grade I complications Blood transfusions and total parenteral nutrition are also included
Grade III IIIa IIIb	Requiring surgical, endoscopic or radiological intervention Intervention not under general anesthesia Intervention under general anesthesia
Grade IV IVa IVb	Life-threatening complication (including CNS complications)* requiring IC/ICU management Single organ dysfunction (including dialysis) Multiorgan dysfunction
Grade V	Death of a patient
Suffix “d”	If the patient suffers from a complication at the time of discharge, the suffix “d” (for “disability”) is added to the respective grade of complication. This label indicates the need for a follow-up to fully evaluate the complication.
